# Comparative connectomics reveals stage-specific gap junction rewiring that reshapes avoidance behavior

**DOI:** 10.64898/2026.05.28.728331

**Published:** 2026-05-29

**Authors:** Daniel T. Choe, David H. Hall, Ken C.Q. Nguyen, Myunghwan Choi, J. Alexander Bae, Junho Lee

**Affiliations:** 1 Department of Biological Sciences, Seoul National University; Seoul, 08826, Republic of Korea; 2 Research Institute of Basic Sciences, Seoul National University; Seoul, 08826, Republic of Korea; 3 Department of Biological Sciences, Korea Advanced Institute of Science and Technology (KAIST); Daejeon, 34141, Republic of Korea; 4 Department of Neuroscience, Albert Einstein College of Medicine; Bronx, NY, 10461, USA

## Abstract

Environmental stress can remodel neural circuits, yet how such rewiring generates behavioral adaptation remains poorly understood. The dauer stage of *Caenorhabditis elegans* provides a unique model to address this question. Here, we investigated how dauer-specific circuit remodeling alters nociceptive behavior. We found that dauers exhibit markedly shorter avoidance durations than adults. Comparative connectomics revealed substantial expansion of gap junctions within the dauer nociceptive circuit. Calcium imaging further showed that dauer neurons exhibit faster and more transient activity dynamics throughout the circuit. Introducing synthetic dauer-specific gap junctions into adults was sufficient to recapitulate dauer-like neuronal activity patterns and significantly shorten avoidance duration. Despite extensive circuit rewiring, however, avoidance initiation remained preserved across developmental stages. Together, our findings demonstrate that the dauer connectome is selectively rewired through gap junction remodeling to tune behavioral persistence while robustly preserving behavioral initiation, revealing how developmental circuit reorganization balances flexibility and stability for survival under stress.

## Introduction

Understanding how differences in neural circuitry give rise to differences in behavior is a central question in neuroscience. *Caenorhabditis elegans* provides a uniquely powerful system for addressing this problem, as it remains the only organism for which connectomes have been reconstructed across all major developmental stages and both sexes (^1^; ^2^; ^3^; ^4^). Among these stages, the dauer represents an alternative developmental form entered under adverse environmental conditions such as food scarcity or elevated temperature (^5^; ^6^). The dauer stage is associated with profound behavioral adaptations that distinguish it from reproductive stages. For example, dauers are attracted to CO_2_, whereas adults typically avoid it (^7^; ^8^); they exhibit faster locomotion (^5^; ^9^); and they display nictation, a specialized dispersal behavior in which worms raise and wave their bodies in three dimensions to promote dispersal (^10^). These qualitative behavioral shifts raise a fundamental question: how does dauer-specific circuit rewiring support stage-specific survival strategies?

The recent reconstruction of the dauer chemical synapse connectome has enabled systematic investigation of how neural circuits are developmentally remodeled under stress conditions and how such remodeling may shape behavior. Network-level analyses revealed that while interneuron-centered connectivity remains largely stable across development, the outputs of sensory neurons are extensively rewired in dauers (^4^). These findings suggest that stage-specific sensory processing may be a major driver of dauer-specific behavioral adaptation and raise the possibility that specific sensory circuits are selectively modified to tune behavioral outputs for survival during developmental arrest.

One well-characterized circuit for addressing this question is the nociceptive circuit, in which ASH sensory neurons relay aversive signals to downstream command interneurons (AVA, AVB, AVD, and PVC), which in turn engage A- and B-type motor neurons to generate escape behavior (^11^; ^12^; ^13^; ^14^; ^15^). In *C*. *elegans*, nociceptive stimuli evoke a stereotyped avoidance response in which animals initiate backward locomotion, reorient, and subsequently resume forward movement. This behavior can be conceptually divided into two components: avoidance initiation, which determines whether an animal begins a reversal in response to a stimulus, and avoidance duration, which determines how long the reversal is maintained before forward locomotion resumes. Together, these components define the temporal structure of nociceptive escape behavior and provide a framework for relating circuit organization to behavioral dynamics. Given the extensive sensory rewiring observed in the dauer connectome, we asked how these components of nociceptive behavior are altered in dauers and how such changes relate to circuit organization and activity. We therefore began by quantitatively comparing nociceptive escape behaviors between dauers and adults, focusing on both avoidance initiation and avoidance duration.

## Results

### Dauers exhibit shortened avoidance duration compared with adults

**Fig. 1:**
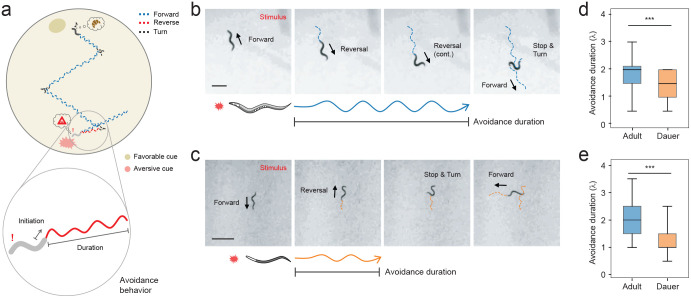
Dauers exhibit shortened avoidance duration compared with adults. **a,** Schematic of the locomotor sequence of *C*. *elegans*. Forward, reversal, and turning movements are indicated by blue, red, and black dashed lines, respectively. Animals initiate reversals in response to aversive stimuli (red) and navigate toward favorable cues (beige). Inset, zoom-in of nociceptive escape behavior, consisting of avoidance initiation and avoidance duration. **b,c,** Representative nociceptive escape behavior in adult (**b**) and dauer (**c**) animals with corresponding schematic representations. Red-light stimulation activates ASH sensory neurons in the first frame. Avoidance duration was defined as the interval from initiation of backward locomotion to resumption of forward movement and quantified as the number of backward sinusoidal body waves generated (blue, adult; orange, dauer). **d,e,** Quantification of avoidance duration in adults and dauers following SDS stimulation (**d**; adults, *n* = 39; dauers, *n* = 40; ***P = 8.3 × 10^−4^) and optogenetic activation of ASH neurons (**e**; adults, *n* = 36; dauers, *n* = 38; ***P = 1.4 × 10^−6^. Statistics: **d,e,** two-sided Mann–Whitney U test.

Freely moving *C*. *elegans* exhibits diverse behavioral sequences consisting of forward movement, reversals, and turns, which allow animals to avoid aversive stimuli and navigate toward favorable cues such as food sources (Fig. 1a). Upon detection of nociceptive signal by ASH sensory neurons, *C*. *elegans* responds through a stereotyped escape sequence in which animals abruptly halt forward locomotion, execute a reversal, and reorient away from the aversive source (Fig. 1a, inset). Since nociceptive avoidance is stochastic (^15^), we define this escape behavior into two components: 1) avoidance initiation, the probability that an animal begins a reversal, and 2) avoidance duration, how long the reversal is maintained before forward locomotion resumes. We first focused on avoidance duration, as differences between dauer and adult animals were immediately apparent. Notably, adult animals appeared to sustain longer reversal behaviors than dauers (Fig. 1b,c), and we therefore quantitatively compared the avoidance duration between two stages.

To quantify avoidance duration, we measured the number of backward sinusoidal body bends generated from reversal onset until forward locomotion resumed (^16^; Fig. 1b–c). This metric was chosen over distance or time because dauers and adults differ in body length and locomotion speed, which would bias direct spatial or temporal comparisons. Using this normalized measure, dauers consistently produced fewer body bends during avoidance than adults, indicating that dauers terminate reversals earlier.

We exposed animals to SDS (sodium dodecyl sulfate), an aversive cue for *C*. *elegans*, and found that dauers exhibit significantly shorter avoidance duration than adults (Fig. 1d). Adults typically generated about two reversal body bends whereas dauers showed about one (Fig. 1d). However, because stronger stimulus concentrations are required to elicit robust reversals in dauers, we asked whether the observed difference might simply reflect unequal sensory drive. To address this, we bypassed sensory input entirely by directly activating ASH neurons optogenetically and again observed a comparable reduction in avoidance duration in dauers (Fig. 1e). Furthermore, avoidance duration remained stable across a range of stimulus intensities within each stage, indicating that avoidance duration is insensitive to stimulus strength in both adults and dauers (Supplementary Fig. 1a). Together, these results demonstrate that shortened avoidance duration in dauers reflects a stage-specific circuit mechanism rather than altered sensory perception.

### Dauer-specific gap junctions rewire the nociceptive circuit

**Fig. 2.**
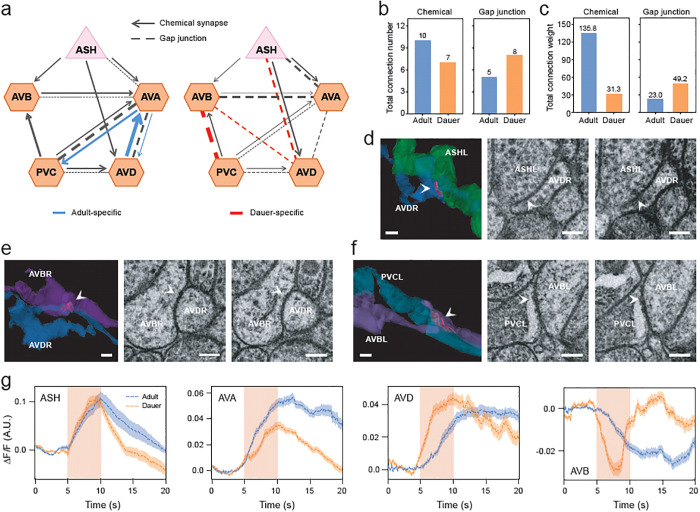
Dauer-specific electrical connections rewire the nociceptive circuit. **a,** Nociceptive circuit diagrams in adult (left) and dauer (right) stages (adult, Cook *et al*., 2019; dauer, Yim *et al*., 2024 and this study). Unless otherwise specified, “adult” refers to the adult hermaphrodite. Adult-specific connections are shown in blue and dauer-specific connections in red. Arrows indicate chemical synapses; dotted lines indicate gap junctions. The thickness of the arrow or dotted line represents the size of the synapse or gap junction (synapse weight follows Cook *et al*^2^ and Yim *et al*^4^). Pink triangles denote sensory neurons and orange hexagons denote interneurons. **b,c,** Quantification of total connection number (**b**) and total connection weight (**c**) of chemical synapses and gap junctions within the nociceptive circuit in adults and dauers. Connection weights were rounded to one decimal place. **d–f,** Representative electron micrographs of dauer-specific gap junctions between ASH and AVD (d), AVB and AVD (e), and AVB and PVC (f), together with three-dimensional reconstructions of the corresponding contact regions. White arrowheads indicate gap junctions in the electron micrographs. Red patches in the three-dimensional reconstructions indicate gap junction contact sites. **g,** Mean calcium activity (ΔF/F ± s.e.m.) of input neurons ASH, AVA, AVD, and the output neuron AVB during ASH stimulation. Shaded region indicates stimulus period. *n* = 15 (ASH dauer), 13 (ASH adult), 35 (AVA dauer), 24 (AVA adult), 35 (AVB dauer), 46 (AVB adult), 61 (AVD dauer), 47 (AVD adult) animals. Scale bar: 200 nm (**d–f**; 3D reconstruction), 100 nm (**d–f**; EM images).

Having established that dauers show shortened avoidance duration through a stage-specific circuit mechanism, we next examined how the nociceptive circuit is structurally different between the adult and dauer stages. Nociceptive circuit has been extensively studied in the context of aversive sensory processing and motor decision-making, making it an ideal substrate for investigating how dauer-specific structural rewiring shapes avoidance behavior (^17^; ^18^; ^19^; ^20^). Nociceptive circuit is composed of the sensory neuron ASH and the command interneurons AVA, AVB, AVD, and PVC. Within this circuit, AVA and AVD promote backward locomotion, with AVA acting as the primary backward command interneuron, while AVB and PVC promote forward locomotion, with AVB playing the dominant role (Fig. 2a).

Comparing adult and dauer nociceptive circuits extracted from published adult and dauer connectomes, we constructed circuit diagrams highlighting the stage-specific connectivity (Fig. 2a; blue: adult-specific, red: dauer-specific). To capture electrical connectivity in the dauer nervous system, we systematically annotated gap junctions—identified as parallel electron-dense membranes separated by a 2–3 nm cleft(^21^; ^22^; ^23^; ^24^)—onto the previously reconstructed chemical synapse map, generating an expanded wiring diagram. As a result, we were able to find that the two stages share a common circuit backbone but differ substantially with additional gap junction connectivity (Fig. 2a). To quantify this reorganization, we compared the total number and weight of chemical synapses and gap junctions within the nociceptive circuits. Chemical connectivity was more dominant in the adult stage as all the adult-specific connections were chemical synapses (Fig. 2a–c). In contrast, gap junction connectivity was markedly expanded in dauer, with both total number and connection weight significantly greater than in adults, as all the dauer-specific connections were gap junctions (Fig. 2a–c).

To confirm that these dauer-specific gap junctions represent bona fide synaptic structures, we examined the relevant contact regions in electron microscopy (EM) images. Dauer-specific electrical connections included gap junctions between ASH and AVD (ASH=AVD; hereafter “=” denotes gap junction) (Fig. 2d), AVB=AVD (Fig. 2e), and AVB=PVC (Fig. 2f). We were able to manually verify clear ultrastructural features of gap junctions — elongated, tight, and uniform apposition between neurons — in EM images for these dauer-specific gap junctions (Fig. 2d–f). Together, these evidences indicate that the dauer nociceptive circuit undergoes genuine structural remodeling through newly formed electrical synapses rather than merely reflecting differences in synapse strength. We next asked whether this rewired nociceptive circuitry reconfigures functional activities of neurons.

### Dauer-specific gap junctions reshape neuronal activity

To determine how dauer-specific circuit rewiring reconfigures neuronal activity, we performed calcium imaging of all neurons within the nociceptive circuit in both adult and dauer animals during optogenetic ASH stimulation. Both adults and dauers showed robust calcium activation in ASH and AVA during stimulation, yet dauer neurons exhibited significantly faster signal decay when the stimulus was removed (Fig. 2g). Dauer AVD neurons showed faster and more transient calcium responses when the stimulus was given, while AVB, which is previously reported to be inhibited by ASH^15^, displayed faster decrease in dauers (Fig. 2g). PVC did not show robust response to ASH stimulation, consistent with previous whole-brain imaging results^25^ (data not shown).

Overall, dauer neurons showed more transient responses across all neurons in the nociceptive circuit, suggesting that the newly formed dauer-specific gap junctions accelerate signal propagation and termination within the circuit. It is interesting to note that AVB, the primary forward command interneuron, exhibited more rapid decrease and recovery from inhibition in dauers, which may directly facilitate earlier transition to forward movement following reversal behavior. Therefore, we further attempted to answer whether dauer-specific gap junctions could produce transient calcium dynamics, leading to dauer-like avoidance behavior.

### AVB-AVD gap junction is necessary for dauer-like neuronal dynamics

**Figure 3.**
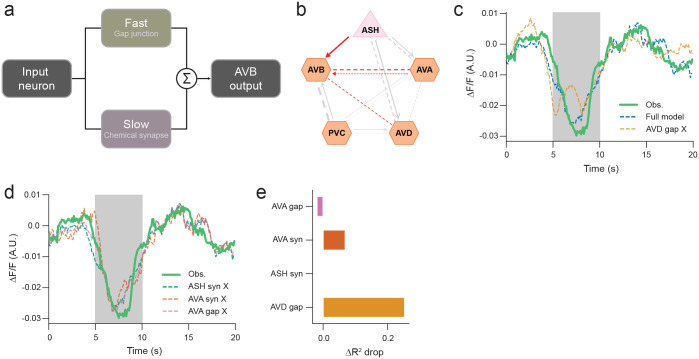
A two-timescale model captures AVB calcium dynamics through dauer-specific gap junction connectivity. **a,** Schematic of the two-timescale model. AVB calcium activity is modeled as a linear combination of fast (gap junction) and slow (chemical synapse) input components from upstream neurons. **b,** Dauer-state neural circuit diagram showing connectivity used for model inputs. Bold red arrow indicates the AVD-AVB gap junction identified as dauer-specific. Solid arrows indicate chemical synapses; dashed lines indicate gap junctions; dashed arrow indicate presumed extrasynaptic connection. Line weight reflects connectivity strength. **c,** The full connectivity-constrained model (blue dashed) accurately reproduces observed AVB activity (green), whereas removal of the AVD gap junction (orange dashed) substantially degrades model fit. **d,** Ablation of other individual synaptic connections — ASH chemical synapse, AVA extrasynaptic connection, and AVA gap junction — does not substantially impair model fit, indicating that these shared connections are not critical for capturing AVB dynamics. **e,** Ablation analysis quantifying the drop in R^2^ (ΔR^2^) upon removal of each synaptic pathway. The AVD gap junction contributes the largest unique variance to the model fit (ΔR^2^ ≈ 0.25), whereas other connections contribute minimally, identifying the AVD-AVB gap junction as a functionally critical dauer-specific connection.

To assess whether dauer AVB calcium dynamics can be explained by connectivity-constrained presynaptic inputs, we modeled AVB activity as a weighted sum of those inputs. Based on the distinct signaling properties of electrical and chemical synapses, we modeled gap junction inputs as a fast component and chemical synapse input as a slow component, resulting in a linear dynamical system with two-timescales^26^ (Fig. 3a; See Methods). Since PVC did not show meaningful responses during ASH stimulation, we constrained the model to three presynaptic inputs: ASH, AVA, and AVD (Fig. 3b).

Fitting the model to the observed mean AVB calcium activity using all connectivity-constrained inputs yielded a strong fit (R^2^=0.807), accurately capturing both the timing of the inhibitory decrease and the subsequent recovery of AVB activity (Fig. 3c). To evaluate specific contribution of the dauer-specific AVB=AVD, we systematically removed this connection and refitted the model. Ablation of the AVB=AVD input substantially degraded model performance (R^2^=0.595), with the model failing to precisely predict the timing of both the decay and recovery phases of AVB activity (Fig. 3c,e). Since a model incorporating all connectivity parameters could perform better simply by virtue of having more free parameters, we performed an additional control in which shared connections between dauer and adult were selectively removed. These models still captured observed AVB dynamics reasonably well (Fig. 3d–e), confirming that the model fit is not solely attributable to the number of parameters. This result indicates that the AVB-AVD gap junction is a necessary component for explaining the transient AVB dynamics observed in dauers, and cannot be compensated for by the remaining presynaptic inputs alone.

### Dauer-specific gap junctions reshape neuronal activity to shorten avoidance duration

**Fig. 4.**
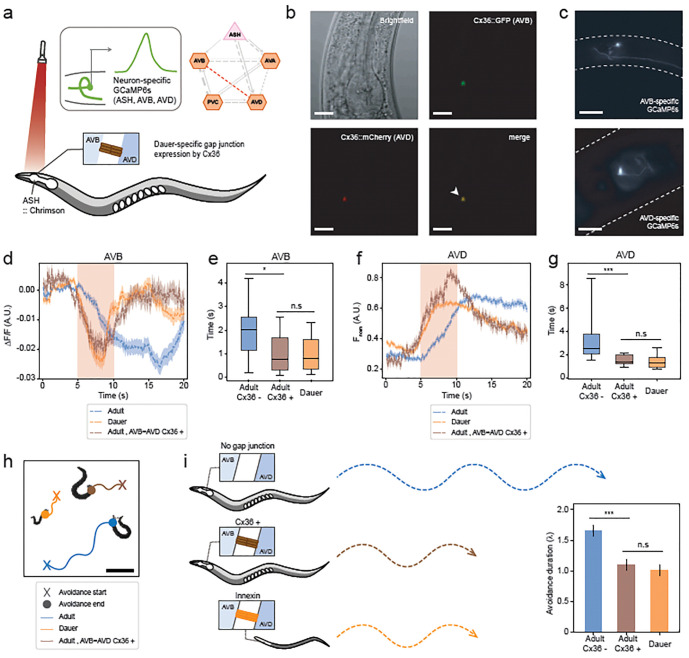
Synthetic AVB=AVD electrical coupling reproduces dauer-like circuit dynamics and behavior. **a,** Schematic of the experimental strategy. Adult transgenic animals expressed a connexin-36 (Cx36)–mediated synthetic gap junction between AVB and AVD together with neuron-specific GCaMP6s reporters and red-light–activated channelrhodopsin (Chrimson) in ASH sensory neurons. Right, position of the dauer-specific AVB=AVD gap junction within the nociceptive circuit (red). **b,** Confocal validation of Cx36 expression in AVB (GFP, green) and AVD (mCherry, red). Brightfield and merged fluorescence images are shown. White arrowhead indicates sites of Cx36-mediated gap junction formation (yellow in merge). **c,** Representative images of neuron-specific GCaMP6s expression in AVB (left) and AVD (right). White dashed line outlines the worm body. **d,f,** Calcium activity traces of AVB (**d**) and AVD (**f**) in adults (blue), dauers (orange), and adults expressing the synthetic AVB=AVD gap junction (brown). Imaging was performed at 100 ms per frame. Red shading indicates stimulation (frames 50–100; 5–10 s). Sample sizes: **d**, *n* = 34 (adult Cx36−), 35 (dauer), 12 (adult Cx36+); **f**, *n* = 44 (adult Cx36−), 61 (dauer), 13 (adult Cx36+). **e,g,** Comparison of response time constants in AVB (**e**) and AVD (**g**). **e**, decay time constant of AVB responses following stimulus onset (*P = 0.0142). **g**, rise time constant of AVD responses following stimulus onset (***P = 1.6 × 10^−5^). **h,** Representative locomotor trajectories during escape behavior in adults (blue), dauers (orange), and adults expressing the synthetic AVB=AVD gap junction (brown). Traces were aligned to stimulus onset and plotted at identical scale (×40). The trajectory from initiation of avoidance following stimulus delivery (marked by ‘X’) to resumption of forward locomotion (marked by ‘O’) is highlighted. Individual trajectories were recorded separately and overlaid onto a common reference frame. **i,** Quantification of avoidance duration. Adults expressing Cx36-mediated AVB=AVD coupling exhibit shortened reversal duration comparable to dauers (*n* = 36 adult Cx36−, 34 adult Cx36+, 27 dauer; ***P = 1.15 × 10^−4^). Statistics: two-sided Wilcoxon rank-sum test (**e,g**); two-sided Mann–Whitney U test (**i**). Scale bars: 20 μm (**b,c**), 0.5 mm (**h**). Error bars: mean ± s.e.m. (**i**).

To test whether the modeling results correctly predicted the importance of the dauer-specific AVB=AVD in shortening avoidance duration, we examined how introducing this connection affects neuronal activity and behavior in animals. We generated transgenic adult strains expressing a synthetic connexin-36–mediated (Cx36-mediated;^27^) gap junction between AVB and AVD, together with neuron-specific GCaMP6s expression in AVB and AVD, allowing direct measurement of activity changes induced by the dauer-specific AVB=AVD connection (Fig. 4a–c). Calcium imaging revealed that activity dynamics in both AVB and AVD shifted toward a dauer-like pattern in Cx36-expressing adults, demonstrating that dauer-specific gap junctions are sufficient to generate dauer-like neuronal dynamics (Fig. 4d,f). Consistent with accelerated circuit transitions, response time constants in both AVB and AVD were significantly reduced in dauers and in adults expressing the dauer-specific gap junction relative to wild-type adults following stimulus onset (Fig. 4e,g). We next asked whether these neuronal changes were sufficient to alter behavior. Adults expressing the AVB=AVD Cx36 exhibited significantly shortened avoidance duration, comparable to that observed in dauers (Fig. 4h–i). Because avoidance duration reflects the persistence of backward command activity and the timing of forward circuit re-engagement, these results support a model in which dauer-specific electrical coupling reshapes circuit dynamics to accelerate the transition back to forward locomotion.

**Fig. 5.**
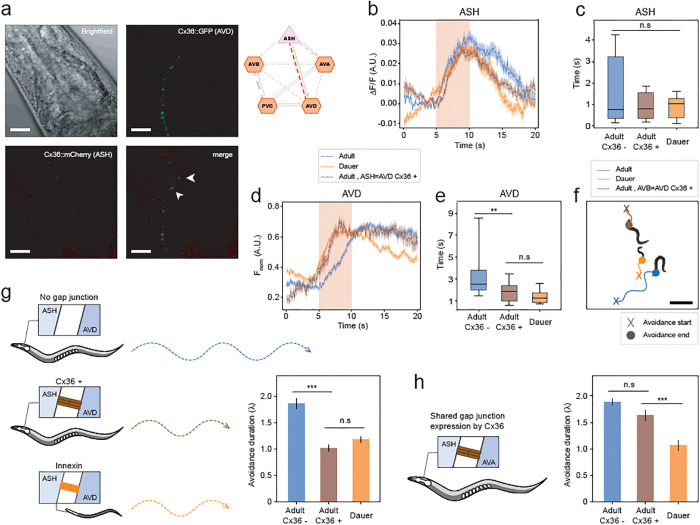
Synthetic ASH=AVD electrical coupling reproduces dauer-like escape dynamics and behavior. **a,** Confocal validation of Cx36 expression in AVD (GFP, green) and ASH (mCherry, red). Brightfield and merged fluorescence images are shown. White arrowheads indicate sites of Cx36-mediated gap junction formation (yellow in merge). Right, position of the dauer-specific ASH=AVD gap junction within the nociceptive circuit (red). **b,d,** Calcium activity traces of ASH (**b**) and AVD (**d**) in adults (blue), dauers (orange), and adults expressing the synthetic ASH=AVD gap junction (brown). Imaging was performed at 100 ms per frame. Red shading indicates stimulation (frames 50–100; 5–10 s). Sample sizes: **b**, *n* = 11 (adult Cx36−), 12 (dauer), 18 (adult Cx36+); **d**, *n* = 44 (adult Cx36−), 61 (dauer), 13 (adult Cx36+). **c,e,** Comparison of response time constants in ASH (**c**) and AVD (**e**). For AVD responses, rise time constants following stimulus onset differed between adult Cx36− and adult Cx36+ (**P = 0.00775). **f,** Representative locomotor trajectories during escape behavior in adults (blue), dauers (orange), and adults expressing the synthetic ASH=AVD gap junction (brown). Traces were aligned to stimulus onset and plotted at identical scale (×40). The trajectory from initiation of avoidance following stimulus delivery (marked by ‘X’) to resumption of forward locomotion (marked by ‘O’) is highlighted. Individual trajectories were recorded separately and overlaid onto a common reference frame. **g,h,** Quantification of behavioral effects of synthetic electrical coupling. Expression of a dauer-specific ASH=AVD gap junction shortened reversal duration to dauer-like levels (**g**; *n* = 30 adult Cx36−, 30 adult Cx36+, 46 dauer; ***P = 2.7 × 10^−8^), whereas expression of a shared ASH=AVA gap junction did not shorten reversal duration (**h**; *n* = 57 adult Cx36−, 57 adult Cx36+, 29 dauer; ***P = 5.34 × 10^−4^). Statistics: two-sided Wilcoxon rank-sum test (**c,e**); two-sided Mann–Whitney U test (**g,h**). Scale bar: 20 μm (**a**), 0.5 mm (**f**). Error bars: mean ± s.e.m. (g,h).

To test whether this effect generalizes across multiple dauer-specific electrical connections, we generated a second transgenic adult strain expressing a synthetic Cx36-mediated gap junction between ASH and AVD, another dauer-specific electrical connection (Fig. 5a). Although ASH decay kinetics were largely unchanged across stages and transgenic adults (Fig. 5b–c), AVD responses shifted toward a dauer-like pattern (Fig. 5d). Time constants of AVD responses were significantly reduced in dauers and in Cx36-expressing adults relative to wild-type adults (Fig. 5e). Avoidance duration was likewise significantly shortened in Cx36-expressing adults (Fig. 5g), further demonstrating that dauer-specific electrical coupling is sufficient to reduce reversal duration. In contrast, introducing a synthetic ASH=AVA gap junction—which differs in strength between stages but is not dauer-specific—did not alter avoidance duration (Fig. 5h), indicating that the behavioral effects are specific to dauer-enriched electrical connectivity patterns. Together, these results indicate that dauer-specific electrical synapses actively reshape neuronal state transitions and behavioral persistence.

### Avoidance initiation is preserved despite extensive circuit rewiring

**Fig. 6.**
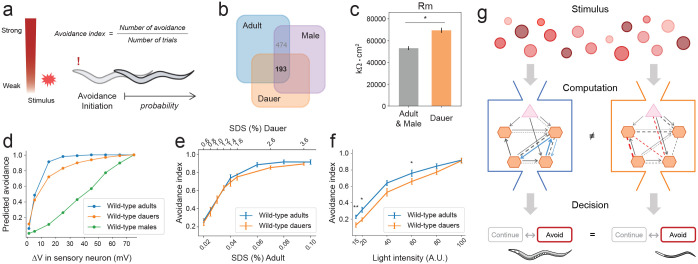
Conserved avoidance initiation despite developmental circuit rewiring. **a,** Schematic of avoidance index calculation. The avoidance index quantifies the probability of initiating backward escape behavior across a range of stimulus intensities. **b,** Venn diagram of valid parameter sets used for network simulations. Of 667 parameter combinations that reproduced avoidance behavior in adult hermaphrodite and male circuits, 193 also satisfied constraints for the dauer circuit (black outline). **c,** Comparison of membrane resistance (Rm) distributions between parameter sets valid for adult hermaphrodite and male circuits (‘Adult & Male’; 677 sets, gray) and those valid for the dauer circuit across the extended Rm range (‘Dauer’; 1,434 sets, orange) (*P = 0.0154). **d,** Simulated avoidance index curves for adult (blue) and dauer (orange) nociceptive circuits. **e,** Avoidance index measured in adults and dauers in response to SDS. Adults were tested across SDS concentrations from 0.02–0.10%, and dauers across 0.6–3.6%, selected to match the dynamic range of adult avoidance responses. Sample sizes (adults): *n* = 16, 13, 13, 13, and 14 for 0.02–0.10%. Sample sizes (dauers): *n* = 12, 12, 25, 22, 12, 12, 15, and 15 for 0.6–3.6%. **f,** Avoidance index measured in adults and dauers following optogenetic activation of ASH expressing ChR2. ATR-fed animals were illuminated with blue light at the indicated intensities (*n* = 15 per group). **g,** Conceptual summary of avoidance initiation in adults and dauers. Despite differences in circuit architecture (middle), adults and dauers exhibit similar avoidance initiation across stimulus conditions (top), resulting in comparable avoidance index profiles (bottom). Statistics: two-sided Wilcoxon rank-sum test (**c**); two-sided Mann–Whitney U test (**f**). Error bars: mean ± s.e.m. (**c,e,f**).

While avoidance duration was strongly altered in dauers, we next asked whether other nociceptive behaviors were similarly affected. We therefore examined the avoidance index, which quantifies how effectively animals initiate avoidance across a range of noxious stimulus intensities (^15^) (Fig. 6a). Because a computational framework for avoidance index simulation had previously been established (^15^; ^28^; ^29^), we adapted this framework to incorporate the dauer circuit architecture. Of the 667 parameter sets previously identified as valid for both hermaphrodite and male circuits, 193 remained valid when the dauer circuit was introduced (Fig. 6b).

Because neuronal processes in dauers are thinner than in adults, passive electrical properties of the circuit are expected to differ. To account for morphological differences between stages, we first adjusted the fixed surface area parameter used to estimate input resistance and sensory input. Using volumetric reconstruction data, we calculated mean neuronal surface areas for dauer and adult stages and proportionally scaled the surface area parameter for the dauer network. Because specific membrane resistance (Rm) reflects intrinsic membrane properties per unit area and is independent of neuronal geometry, we next asked whether avoidance initiation remains robust across a broader range of physiologically plausible Rm values. We therefore selectively expanded the membrane resistance parameter space beyond values represented in the previously validated parameter sets, while maintaining experimentally constrained relationships among other circuit parameters. This targeted expansion allowed us to assess whether preserved avoidance behavior remains stable under variation in intrinsic membrane properties distinct from morphological scaling. Extending membrane resistance values yielded 1,434 valid parameter sets for the dauer network, which exhibited significantly higher Rm distributions (Fig. 6c).

Using these adjusted parameter sets, simulated avoidance index curves for the dauer closely resembled those of adults, producing similar logarithmic response profiles with only minor differences in the early response slope (Fig. 6d), indicating that avoidance initiation remains robust across a wide range of passive membrane properties. Behavioral measurements confirmed these predictions. Because dauers possess a sealed buccal cavity (^30^; ^31^), we measured avoidance index across a broader range of SDS concentrations and compared curves after matching avoidance index values across developmental stages. Dauers exhibited avoidance curves nearly identical to those of adults (Fig. 6e). Optogenetic activation of ASH likewise produced comparable avoidance indices between dauers and adults (Fig. 6f). Together, these results indicate that avoidance initiation remains preserved across developmental stages despite extensive circuit rewiring (Fig. 6g).

## Discussion

Neural circuits can undergo substantial developmental remodeling that alters behavior while preserving core functional outputs. Here, we show that rewiring of the dauer nociceptive circuit selectively modifies the temporal dynamics of avoidance duration while leaving avoidance initiation largely intact. Dauers terminate avoidance more rapidly and resume forward locomotion sooner than adults (Fig. 1b–e), and this behavioral difference is accompanied by distinct interneuron activity patterns that favor rapid reinstatement of forward movement (Fig. 2g). Importantly, introducing dauer-specific gap junctions into adults was sufficient to reproduce both dauer-like neuronal dynamics and shortened avoidance duration, supporting a model in which stage-specific electrical coupling contributes to the timing of circuit responses (Fig. 4,5). Comparative analysis of dauer and adult connectomes further revealed that binary dimorphic chemical synapses present in adults are absent in dauers, whereas developmental rewiring occurs predominantly through gap junctions (Fig. 2a). At the same time, chemical synaptic connectivity is broadly weakened in the dauer circuit (Fig. 2b,c). Together, these findings suggest a developmental strategy in which electrical coupling supplements or partially replaces chemical transmission, preserving essential behavioral outputs while modifying the temporal structure of circuit activity. More specifically, our results are consistent with a model in which electrical coupling redistributes signal flow within the circuit, accelerating recruitment of forward-promoting pathways and thereby shortening reversal states.

Because the dauer stage is characterized by severe energetic constraints, one possible interpretation is that its circuit architecture reflects adaptations that improve signaling efficiency. Chemical synaptic transmission is metabolically demanding, requiring continuous vesicle recycling and neurotransmitter turnover, whereas gap junctions provide a comparatively efficient mode of signal transmission (^32^; ^33^). Increased electrical coupling may therefore reduce the energetic cost of sustained synaptic activity while limiting the neural resources required for prolonged backward locomotion. In this view, developmental rewiring through electrical synapses could enable efficient escape from aversive stimuli while minimizing energy expenditure during prolonged developmental arrest.

This energetic perspective may also help explain how the dauer nervous system preserves functional integrity despite developmental quiescence. Previous connectomic analyses showed that the overall size of the dauer chemical-synapse network remains comparable to that of adults, indicating that large-scale neural development proceeds even under energetically challenging conditions (^4^). Gene expression data further show that transcripts encoding gap junction components peak during early developmental stages, when electrical coupling is thought to scaffold initial circuit assembly (^34^; ^35^; ^36^; ^37^; Supplementary Fig. 2a–h). Our findings raise the possibility that dauers retain a subset of developmentally transient gap junctions—normally pruned during maturation—throughout quiescence while maintaining an adult-like chemical connectome. Such persistence of electrical coupling could enhance the efficiency of chemically mediated signaling, allowing the dauer nervous system to remain both responsive and metabolically economical. Future comparative connectomes of earlier larval stages could test whether dauer-specific gap junctions overlap with early developmental electrical connections, whereas reconstruction of post-dauer adults could determine whether these connections are selectively removed once reproductive development resumes. Together, these approaches may clarify how reversible developmental rewiring through electrical synapses contributes to the resilience and plasticity of the *C*. *elegans* nervous system.

Beyond circuit connectivity, intrinsic neuronal properties may also contribute to the functional robustness of the dauer nervous system. Because specific membrane resistance (Rm) reflects intrinsic membrane properties per unit area, such as ion channel density and membrane leak, it is distinct from geometric factors including neurite size and surface area. Our simulations show that avoidance initiation remains robust across an expanded range of Rm values (Fig. 6d; Supplementary Fig. 3a–f), suggesting that this behavior is resilient to variation in intrinsic membrane properties as well as circuit architecture. Notably, the broader Rm distributions observed in parameter sets consistent with the dauer circuit are compatible with a regime of reduced effective membrane leak, which would increase neuronal input resistance and enhance voltage responses to synaptic input. Such changes could promote efficient signal propagation under energetically constrained conditions while maintaining reliable initiation of escape behavior. Although direct measurements of membrane properties in dauers will be required to test this possibility, these results raise the possibility that developmental remodeling involves coordinated adjustments of both circuit connectivity and intrinsic neuronal properties to preserve core behavioral functions.

Despite extensive circuit remodeling, avoidance initiation remained remarkably stable. Both behavioral measurements and simulations showed that the avoidance index in dauers closely matches that of adults, indicating that developmental rewiring does not compromise the ability to initiate avoidance responses (Fig. 6d–f). Although motor neuron connectivity was not available in the dauer connectome, simulations using alternative motor connectivity produced similar outcomes, indicating that preservation of avoidance initiation primarily depends on sensory-to-interneuron organization rather than downstream motor wiring (Supplementary Fig. 4a,b). Maintaining robust avoidance initiation is likely critical for dauer survival. Because dauers possess a sealed buccal cavity and therefore limited sensory access to the environment, stimuli that reach the nervous system must reliably trigger rapid avoidance responses.

In summary, our study demonstrates how developmental circuit rewiring can balance adaptive flexibility with circuit stability. By introducing stage-specific electrical coupling while preserving a shared core circuit architecture, the dauer nervous system selectively modifies the temporal dynamics of avoidance behavior while maintaining robust behavioral initiation. Notably, similar behavioral outputs can arise from distinct internal circuit dynamics, indicating that developmental plasticity can retune how a circuit operates without altering what it computes (Fig. 6g;^38^). More broadly, our results suggest that developmental rewiring in this system preferentially targets circuit dynamics rather than core input–output relationships, allowing behavioral timing to adapt while preserving functional reliability. These findings highlight electrical synapses as versatile modulators of circuit function and establish the dauer as a model for understanding how nervous systems reconcile developmental plasticity with robust behavioral output.

### Limitations

A caveat concerns gap junction annotation. Although our dauer dataset includes complete gap junction reconstructions, identifying these synapses in EM images is inherently challenging due to staining variability, oblique membrane angles, and the subtle ultrastructure of gap junctions (^39^; ^40^). Accurate annotation requires extensive training, and thus caution is warranted when interpreting the full gap junction network at the whole-brain level. For the nociceptive circuit, we validated dauer-specific gap junctions against an independent, unpublished dataset and confirmed their presence symmetrically across left–right homologs, supporting their reliability. Nevertheless, for other circuits of interest, similar cross-dataset confirmation will be essential. Future advances in EM imaging or staining techniques that selectively highlight gap junctions will further strengthen the accuracy of comparative connectomic analyses and enable more robust network-level simulations (^41^; ^42^; ^43^; ^44^).

## Methods

### Experimental model and subject details

*C. elegans* were cultured and handled at 20°C according to standard methods(^45^). *C. elegans* Bristol strain N2 and JN2389 (peIs1090[*sra-6*p::ChR2Y2]) lines were used, obtained from the Caenorhabditis Genetics Center (CGC).

### Dauer induction

For all behavioral assays and imaging experiments, seven to twelve young adult hermaphrodites (depending on the growth rate of each strain or transgenic line) were transferred to synthetic pheromone plates to induce dauer formation. Plates contained 10 g/L agar, 7 g/L agarose, 2 g/L NaCl, 3 g/L KH_2_PO_4_, 0.5 g/L K_2_HPO_4_, 8 mg/L cholesterol, and 2 mg/L synthetic pheromone (ascaroside 2), and were seeded with *Escherichia coli* OP50. Animals were maintained at 25 °C for 4 days, after which dauers were identified based on morphological criteria, including radial body constriction and reduced body size. To ensure that only dauers were selected, plates were treated with 1.0% SDS for 30 minutes. Surviving animals were washed off with an M9 buffer, transferred to unseeded plates, and allowed to recover for at least 30 minutes prior to use.

### Gap junction annotation

Gap junctions were annotated using Neuroglancer while blinded to cell IDs. Because gap junctions are subtle features in EM datasets, a single experienced annotator performed the entire annotation to ensure consistency. Gap junctions were marked with an annotation line connecting the two neurons involved, and the annotation was extended across all consecutive z-sections in which the junction appeared. Annotation data were then used to assign synaptic partners based on the cell IDs of the connected neurons.

Annotations that did not link two distinct neurons, or that linked a neuron to itself, were discarded. In cases where multiple annotations for the same neuronal pair were present in a given z-section and their coordinates fell within 100 nm of each other, only one annotation was retained.

### Avoidance index: tail-drop repellent assay

Avoidance assays were performed as previously described(^46^; ^14^; ^15^), with minor modifications using N2 strain. Adult-stage experiments were conducted on young or 1-day-old adults, and dauer-stage experiments on 5–6 day dauers induced on pheromone plates (hereafter, “dauer” and “adult” refer to hermaphrodite dauer and hermaphrodite adult unless otherwise specified). Prior to testing, animals were transferred to foodless NGM lite plates and allowed to habituate for 10 min. Each animal then underwent 10 repellent stimulations, separated by at least 2 min.

Stimuli consisted of a small drop of sodium dodecyl sulfate (SDS) applied near the tail of a forward-moving animal using hand-pulled capillaries mounted on a mouth-controlled holder. Because dauers often remain motionless, they were gently aroused by tapping the plate until head movement was observed before stimulation. Assay plates were prepared the day before by drying unseeded NGM lite plates for 2 h at 37 °C and leaving them overnight at room temperature. Responses were scored in a 4 s window as reversal (1) or no reversal (0), and the avoidance index for each animal was calculated as the fraction of reversals across 10 trials. For adults, SDS concentrations ranged from 0.02% to 0.10%, while for dauers, concentrations from 0.10%—the lowest dose eliciting consistent responses—to 3.6% were tested.

### Avoidance index: optogenetic stimulation

Optogenetic assays were performed using strain JN2389, which expresses channelrhodopsin-2 (ChR2) in ASH neurons. This strain carries a mutation in *lite-1*, ensuring that behavioral responses reflect ChR2 activity rather than endogenous blue-light receptors. Experimental groups were raised on plates (pheromone plates for dauer induction) seeded with OP50 supplemented with 100 μM all-trans-retinal (ATR). Control groups were raised without ATR. All plates were handled in the dark to prevent ATR degradation. Young or 1-day-old adults were used for adult-stage assays, and dauers induced for 5–6 days were used for dauer-stage assays.

Animals were transferred to unseeded NGM lite plates and habituated for 10 min before testing. Experiments were performed at 22 °C using a Leica M205 FA fluorescence microscope to image and deliver light stimulation. Only animals showing forward movement before light stimulus were used for testing. During each trial, animals were exposed to a 2 s light pulse, and reversals were scored within a 3 s window (stimulus period plus one additional second). Responses were scored as reversal (1) or no reversal (0). Each animal was tested in 10 trials with ≥2 min intervals between stimuli. Data from animals lost before completing 10 trials were excluded. The avoidance index was calculated as the mean fraction of reversals per animal.

Light intensity was varied by adjusting the laser source to six calibrated levels: 20% of 75% maximum, 20% of 100% maximum, 40% of 100% maximum, 60% of 100% maximum, 80% of 100% maximum, and 100% of 100% maximum. These corresponded to relative artificial units (A.U.) of 15, 20, 40, 60, 80, and 100, respectively.

### Avoidance duration: optogenetic stimulation

Avoidance durations were defined as the number of sinusoidal body bends executed during backward locomotion before worms resumed forward movement. To measure this under optogenetic stimulation, we used strain JN2389 or derivative transgenic lines carrying connexin36-mediated synthetic gap junctions. The basic setup for optogenetic stimulation, including growth conditions and age-matched stages for adults and dauers, was identical to the avoidance index assay.

Animals were transferred to unseeded NGM lite plates, habituated for 10 min, and then exposed to 4 s of light stimulation. The number of sinusoidal body bends was counted manually until the animal initiated forward movement. Based on the initial posture and head orientation at stimulus onset, reversal waves were quantified using the following function:

$$f\left(t\right)=\frac{n+1}{2},n\pi <t\leq \left(n+1\right)\pi \;\left(n=0,\;1,\;2,\;3,\cdots \right)$$

where *t* denotes how much a worm moved in sine waves. A score of 0 was assigned to animals that continued moving forward throughout stimulation or that transiently paused but resumed forward movement without executing a reversal. For measuring reversal waves in adults expressing connexin36-mediated gap junctions, experimental and control groups were always assayed on the same day, with blind labeling performed by an independent experimenter.

Some animals expressing connexin36-mediated synthetic gap junctions were developmentally arrested. To ensure consistent physiological comparisons, only fully developed adults carrying ectopic gap junctions—and showing no overt locomotion defects—were included in the analysis of reversal waves in adults expressing connexin36-mediated gap junctions.

### Calcium imaging

Transgenic lines in an N2 background, expressing Chrimson in ASH neurons and GCaMP6s in specific target neurons, were immobilized on 10% agarose pads prepared with a suspension of 0.10 μm microspheres (Polybead^®^, Cat# 00876–15) and sealed with a coverslip. This method rendered worms immobile throughout imaging. Calcium imaging was performed on a Nikon Eclipse Ti2-E inverted microscope equipped with a 40× water-immersion objective. Red LED light (Thorlabs, Solis 623c) was used for Chrimson stimulation, and blue LED light (Thorlabs, Solis 445c) was used for GCaMP6s excitation. Fluorescence was collected through a CFP excitation filter (Chroma Technology, AT440DC, λ = 420–442 nm) and a CFP emission filter (Chroma Technology, ET460/30x, λ = 440–480 nm). Blue light (100 mA) was continuously applied to measure baseline and inhibitory signals, while red LED stimuli (8,000 mA) were delivered between 5–10 s manually after recording onset for a duration of 5 s. Images were acquired at 100 ms per frame for 200 frames, yielding a total recording period of 20 s. Files in which animals moved during acquisition were discarded.

Raw nd2 files were processed using a custom Python script built on the nd2 library. For each recording, regions of interest (ROIs) were manually defined to capture neuronal somata across all frames. Fluorescence intensity was calculated as the mean pixel value within each ROI. Baseline fluorescence (F_0_) was determined as the average intensity during the 50 frames (5 s) preceding stimulus onset. For each time point, *Δ*F was calculated by subtracting F_0_ from the raw value, and the normalized response (*Δ*F/F_0_) was obtained by dividing by F_0_ to account for inter-animal variability in baseline fluorescence. All statistical analyses were performed on normalized data. For peak response comparisons, the maximum *Δ*F/F_0_ value following stimulation was used; for average intensity comparisons, mean signal strength during specified time windows was analyzed.

### Calcium signal fitting

Calcium dynamics were quantified by fitting mono-exponential functions to fluorescence traces. Signal decay and rise were modeled using the following equations, respectively:

(1) $$F\left(t\right)=F_{0}+A\cdot exp\left(-t/\tau \right)$$

(2) $$F\left(t\right)=F_{0}+A\cdot exp\left(t/\tau \right)$$

where *F(t)* denotes fluorescence at time *t*, *F*_*0*_ is baseline fluorescence, *A* is amplitude, and *τ* is the time constant. Traces with excessive noise were smoothed by averaging over a 10-frame sliding window prior to fitting to obtain stable estimate of *τ*. Decay constants were measured in ASH neurons following the end of a 5-s stimulus and in AVB neurons following stimulus onset. Rise constants were measured in AVA and AVD neurons following stimulus onset. Calcium signals from AVD neurons were normalized using min–max scaling prior to fitting.

### Linear dynamic modeling of AVB calcium activity

Using calcium activity recordings during ASH stimulation, we constructed a two-timescale linear dynamical model to model AVB activity based on other presynaptic input neurons’ activities. The model represents the calcium activity of AVB neuron as a weighted sum of fast (electrical synapse) and slow (chemical synapse) components from each presynaptic input as below.

$$x_{AVB}\left(t\right)=\sum_{i}a_{i}\cdot x_{i}\left(t\right)+\sum_{i}b_{i}\cdot y_{i}\left(t\right)$$

$$\frac{{dy}_{i}}{dt}=-\frac{1}{\tau_{i}}y_{i}+x_{i}$$

where $x_{i}\left(t\right)$ is the measured mean calcium activity of presynaptic neuron *i*, *a*_*i*_ is the fast coupling weight representing gap junction input, *b*_*i*_ is the slow coupling weight representing chemical synapse input, $\tau_{i}$ is the integration time constant of the slow component, and $y_{i}\left(t\right)$ is the leaky-integrated copy of the presynaptic input. The slow component was numerically integrated using exact exponential integration for stability.

Connectivity constraints were derived directly from the dauer connectome. For AVB, three presynaptic inputs were considered — ASH, AVA, and AVD — with PVC excluded as it did not show robust calcium responses during ASH stimulation. Constraints were enforced as follows: ASH has no gap junction connection to AVB ($a_{ASH}=0$), and AVD has no chemical synapse onto AVB ($b_{AVD}=0$), yielding six free parameters ($a_{AVA},\;a_{AVD},\;b_{ASH},\;\tau_{ASH},\;b_{AVA},\;\tau_{AVA}$).

Model parameters were estimated by minimizing the mean squared error between the predicted and observed mean calcium traces using differential evolution optimization. Parameter bounds were set to [−2, 2] for coupling weights and [1, 100] frames for time constants. Model performance was evaluated using the coefficient of determination (R^2^).

To assess the specific contribution of individual connections, we performed systematic ablation analysis by setting the coupling weight of each connection to zero and refitting the remaining parameters. The contribution of each connection was quantified as the drop in R^2^ (ΔR^2^) relative to the full connectome-constrained model. We tested ablation of the AVD gap junction, AVA gap junction, ASH chemical synapse, and AVA chemical synapse.

### Plasmid construction and transgenesis

All constructs generated in this study were assembled using the HiFi DNA Assembly Cloning Kit (E5520; New England Biolabs). For constructs involving the Cre/loxP system, promoters and coding sequences were cloned into the pEM1 (loxP; Addgene plasmid #24034) and pEM3 (Cre; Addgene plasmid #24033) vectors as appropriate. Promoter sequences were either amplified from N2 genomic DNA or obtained as gifts from Kyuhyung Kim and Myung-Kyu Choi. GFP and mCherry were obtained from GFP vector (pPD95.77) or mCherry vector (pPD117.01). Detailed information on assembled constructs and promoter pairs is provided in Supplementary Data.

### Transgenic strain generation

Transgenic animals were generated by microinjecting plasmid constructs into the gonads of young adult hermaphrodites using standard procedures. To isolate transgenic progeny, the following co-injection markers were used at the indicated concentrations: *unc-122*p::mCherry (25 ng/μl; red fluorescent coelomocytes), *unc-122*p::GFP (20–25 ng/μl; green fluorescent coelomocytes), *myo-2*p::mCherry (5 ng/μl; red fluorescent pharynx), and *opt-2*p::mCherry (20 ng/μl; red fluorescent intestine). Plasmid DNAs were extracted and purified using a commercial plasmid purification kit prior to injection. Full details of transgenic strains are provided in Supplementary Data.

### Fluorescence microscopy

Images of transgenic worms were acquired using a ZEISS LSM700 confocal microscope (Carl Zeiss). For imaging, worms were paralyzed with 3 mM levamisole and mounted on 3% agar pads. Confocal image stacks were processed using python to merge multiple image layers of the field of interest.

### Chemical synapse connectivity for network simulations

Original connectivity data for adult hermaphrodites and males were taken from Cook *et al*.(^2^), consistent with the datasets used in Pechuk *et al*(^15^). For dauers, chemical synapse strengths were originally quantified differently from the adult dataset and thus required adjustment. To reconcile the two, we used the normalized adult chemical synapse connectivity matrix from Yim *et al*.(^4^), which was processed by averaging synaptic connections between left–right homologous neurons and treating them as a single cell. Scalar factors were then calculated for each connection as follows:

$$\text{scalar}\;{\text{factor}}_{i,j}=\frac{\text{original}\;{\text{matrix}}_{i,j}}{\text{normalized}\;{\text{matrix}}_{i,j}},0\leq i,j\leq 4$$

Of the 13 scalar values obtained, six were identical, each with a value of 4.755 (rounded to the fourth decimal place). This factor was therefore applied uniformly to the dauer chemical synapse connectivity matrix for all network simulations. (Here, 0 ≤ *i,j* ≤ 4 correspond to the indices of a 5 × 5 connectivity matrix representing the neurons ASH, AVA, AVD, AVB, and PVC.) Connectivity between interneurons and A- and B-type motor neurons was adopted from the original adult hermaphrodite connectome. To test the effect of altered inter-to-motor neuron wiring in the dauer network, corresponding connections were replaced with those from the adult male connectome.

### Gap junction connectivity for network simulations

Connection weights between neuron pairs were calculated as the sum of all synaptic sizes observed between them. For gap junctions, connection strength was quantified as the number of consecutive z-sections in which the junction appeared, following the same approach used to assign connection weights in the adult connectome. All gap junction weights were incorporated into the 180 × 180 common connectivity matrix framework previously established in Yim *et al*(^4^). For comparative analyses of the nociceptive circuit, gap junctions between left–right homologous neurons were averaged and treated as a single connection, yielding a 5 × 5 connectivity matrix consistent with that used for chemical synapses. These processed matrices were then used as input for the simulations described below. Gap junction connectivity between interneurons and A- and B-type motor neurons was likewise adopted from the adult hermaphrodite connectome. To test the effect of altered inter-to-motor neuron wiring in the dauer network, these connections were replaced with the corresponding connectivity from the adult male connectome.

### Network simulations

All network simulations were performed using the pipeline developed by Pechuk *et al*. (^15^), with minor modifications to incorporate the dauer nociceptive circuit. Because connectivity to motor neurons was not included in the dauer dataset, adult-stage motor neuron connectivity was substituted for both chemical synapses and gap junctions, yielding a 7 × 7 connectivity matrix. To assess the impact of alternative motor neuron connectivity, we additionally substituted the corresponding male motor neuron connectivity for both connection types.

Parameter sets were drawn from the 667 valid combinations identified by Pechuk *et al*. (^15^) from 15,647,317 (7^7^ × 19) tested combinations, which satisfied both behavioral and physiological constraints in adult hermaphrodites and males (a kind gift from Meital Oren-Suissa). Because dauer neurons exhibit reduced neurite diameters, we adjusted the fixed surface area parameter based on average surface area values calculated from three-dimensional mesh and skeleton data derived from EM reconstructions (Adult: 15 × 10^−6^ cm^2^; Dauer: 12 × 10^−6^ cm^2^). To evaluate applicability to the dauer circuit, we applied the same behavioral and physiological constraints to the dauer network, yielding 193 valid parameter sets. These validated parameter sets were used for all subsequent simulations and analyses. For the extended membrane resistance analysis in the dauer circuit, we evaluated 4,669 parameter sets (667 × 7), corresponding to seven possible membrane resistance values ranging from 15 to 1,500 kΩ·cm^2^, equally spaced on a logarithmic scale. This analysis yielded 1,434 valid parameter sets for the dauer network.

### Statistics

All statistical analyses were performed using custom Python scripts built with the SciPy library. Two-sided Mann–Whitney U tests were used to compare distributions of reversal durations (Fig. 1, Fig. 4, Fig. 5) and two-sided Wilcoxon rank-sum tests were used to compare distributions of time constants and simulated parameters (Fig. 4, Fig. 5, Fig. 6). Statistical analyses for reversal durations were restricted to animals that exhibited a reversal response. Sample sizes and exact *P* values are reported in the corresponding figure legends.

## Supplementary Material

1

## Data Availability

As noted in the Limitations section, certain gap junction annotations of interest require further cross-validation. For this reason, the complete dataset of dauer gap junction connectivity is not included with this publication. However, preliminary annotated synaptic connectivity data are available from the corresponding author upon request.

## Code availability

All analysis code used in this study is publicly available at https://github.com/conncslab/dauer-nociception.
