# Supplementary material for: Comparative connectomics reveals stage-specific gap junction rewiring that reshapes avoidance behavior
Source: bioRxiv. 2026 May 29:2026.05.28.728331. Preprint. [Version 1] doi: 10.64898/2026.05.28.728331 (PMC13232119; doi:10.64898/2026.05.28.728331)
Supplement: 1 [file NIHPP2026.05.28.728331V1-supplement-1.pdf]

a

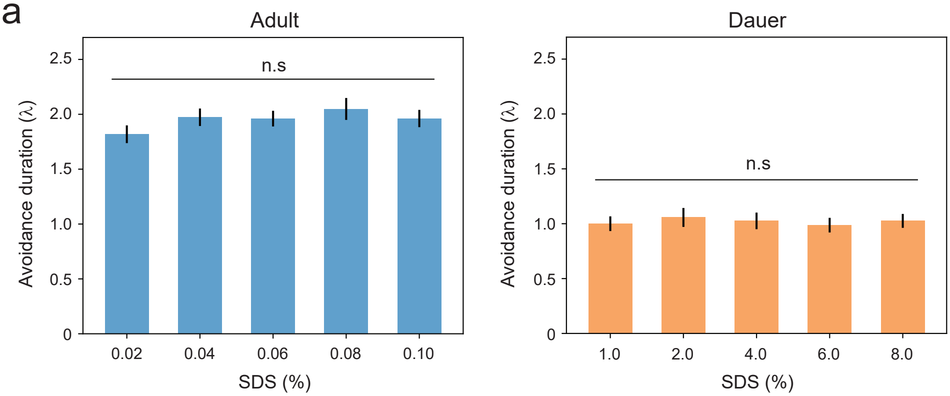
Adult
n.s
Avoidance duration (λ)
2.5
2.0
1.5
1.0
0.5
0
0.02
0.04
0.06
0.08
0.10
SDS (%)
Dauer
n.s
Avoidance duration (λ)
2.5
2.0
1.5
1.0
0.5
0
1.0
2.0
4.0
6.0
8.0
SDS (%)

bioRxiv preprint doi: https://doi.org/10.64898/2026.05.28.728331; this version posted May 29, 2026. The copyright holder has placed this preprint (which was not certified by peer review) in the Public Domain. It is no longer restricted by copyright. Anyone can legally share, reuse, remix, or adapt this material for any purpose without crediting the original authors.

Supplementary Fig. 1. Avoidance duration is invariant across stimulus strengths within each developmental stage.

a, Avoidance duration across stimulus strengths for adults (left; *n* = 33, 37, 38, 41, 39 for 0.02–0.10% SDS) and dauers (right; *n* = 40, 35, 39, 38, 39 for 1.0–8.0% SDS).

Statistics: **a** two-sided Mann–Whitney U test.

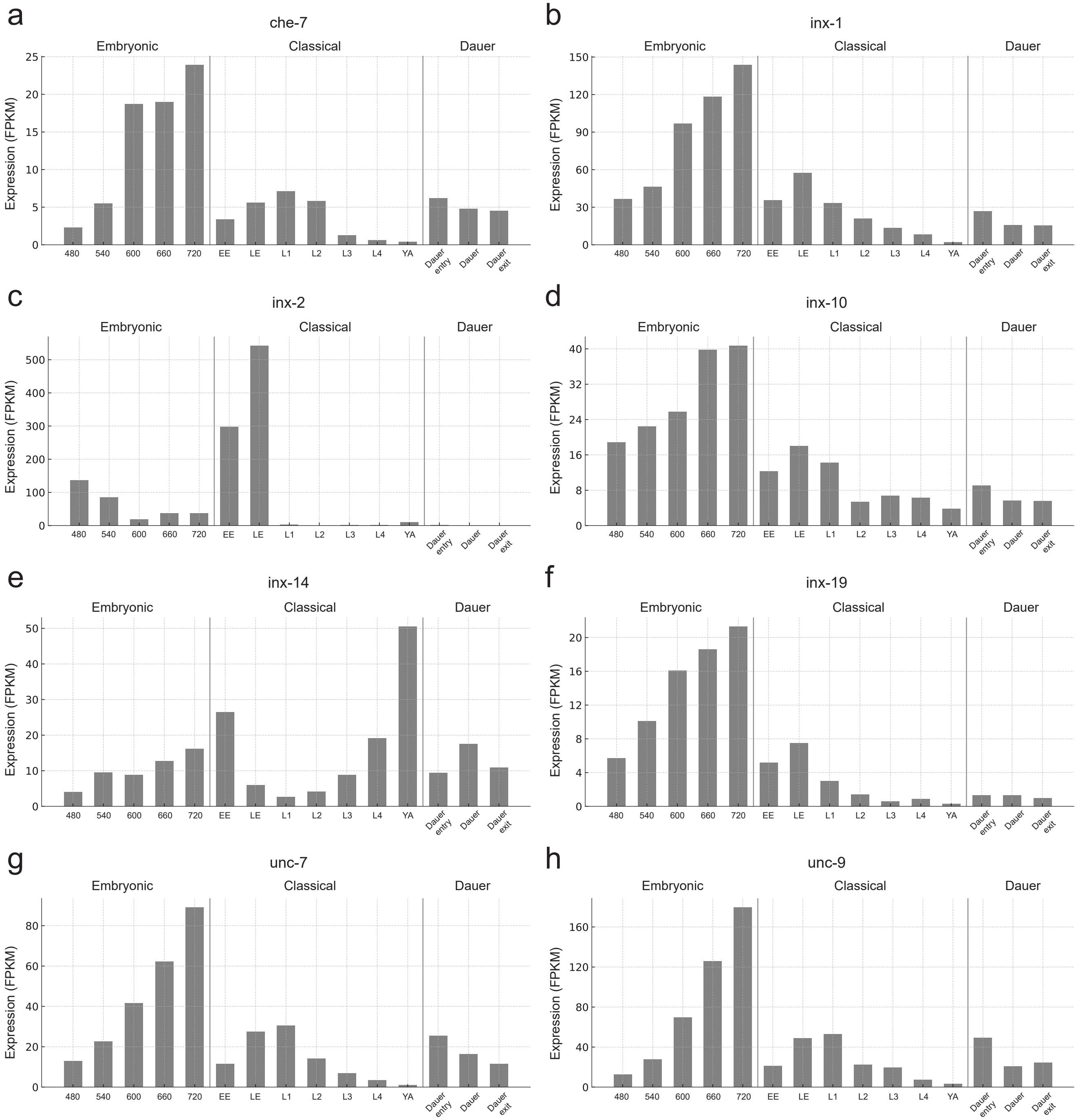
a
che-7
b
inx-1
c
inx-2
d
inx-10
e
inx-14
f
inx-19
g
unc-7
h
unc-9
Embryonic
Classical
Dauer
Expression (FPKM)
480
540
600
660
720
EE
LE
L1
L2
L3
L4
YA
Dauer entry
Dauer
Dauer exit

bioRxiv preprint doi: https://doi.org/10.64898/2026.05.28.728331; this version posted May 29, 2026. The copyright holder has placed this preprint (which was not certified by peer review) in the Public Domain. It is no longer restricted by copyright. Anyone can legally share, reuse, remix, or adapt this material for any purpose without crediting the original authors.

Supplementary Fig. 2. Innexin expression throughout development.

a–h Developmental expression profiles of innexin genes. Bar plots show median expression levels (FPKM) of (**a**) *che-7*, (**b**) *inx-1*, (**c**) *inx-2*, (**d**) *inx-10*, (**e**) *inx-14*, (**f**) *inx-19*, (**g**) *unc-7*, and (**h**) *unc-9* across developmental stages (embryonic stage, classical stage, and dauer stage). These genes were selected as examples expressed in ASH, AVB, or AVD neurons. Expression values are from WormBase annotations.

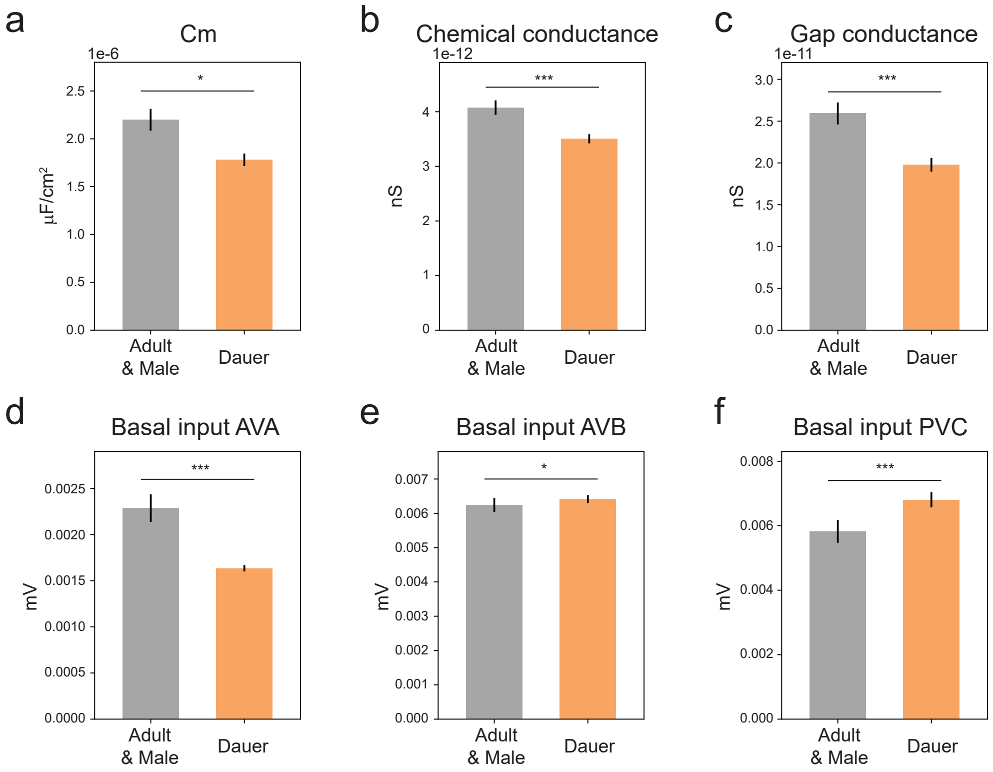

a
Cm
1e-6
μF/cm²
2.5
2.0
1.5
1.0
0.5
0.0
*
Adult & Male
Dauer
b
Chemical conductance
1e-12
nS
4
3
2
1
0
***
Adult & Male
Dauer
c
Gap conductance
1e-11
nS
3.0
2.5
2.0
1.5
1.0
0.5
0.0
***
Adult & Male
Dauer
d
Basal input AVA
mV
0.0025
0.0020
0.0015
0.0010
0.0005
0.0000
***
Adult & Male
Dauer
e
Basal input AVB
mV
0.007
0.006
0.005
0.004
0.003
0.002
0.001
0.000
*
Adult & Male
Dauer
f
Basal input PVC
mV
0.008
0.006
0.004
0.002
0.000
***
Adult & Male
Dauer

bioRxiv preprint doi: https://doi.org/10.64898/2026.05.28.728331; this version posted May 29, 2026. The copyright holder has placed this preprint (which was not certified by peer review) in the Public Domain. It is no longer restricted by copyright. Anyone can legally share, reuse, remix, or adapt this material for any purpose without crediting the original authors.

Supplementary Fig. 3. Parameter distributions associated with the dauer circuit differ from those of adult and male circuits.

Comparison of parameter distributions between sets valid for adult hermaphrodite and male circuits (‘Adult & Male’; 677 sets, gray) and those valid for the dauer circuit across the extended Rm range (‘Dauer’; 1,434 sets, orange). Parameters include membrane capacitance (Cm; **a**), chemical synaptic conductance (**b**), gap junction conductance (**c**), and basal inputs to AVA (**d**), AVB (**e**), and PVC (**f**) (*$P = 0.0140$, ***$P = 3.79 \times 10^{-4}$, *$P = 1.70 \times 10^{-7}$, ***$P = 2.77 \times 10^{-5}$, *$P = 0.0186$, ***$P = 2.79 \times 10^{-6}$, respectively).

Statistics: **a–f,** two-sided Wilcoxon rank-sum test.

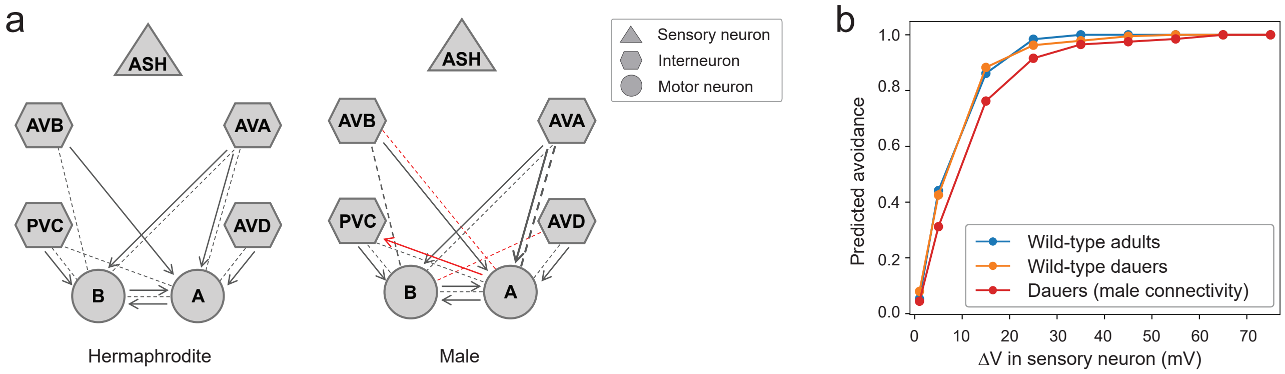

a
ASH
AVB
AVA
PVC
AVD
B
A
Hermaphrodite
ASH
AVB
AVA
PVC
AVD
B
A
Male
Sensory neuron
Interneuron
Motor neuron
b
1.0
0.8
0.6
0.4
0.2
0.0
Predicted avoidance
0
10
20
30
40
50
60
70
ΔV in sensory neuron (mV)
Wild-type adults
Wild-type dauers
Dauers (male connectivity)

bioRxiv preprint doi: https://doi.org/10.64898/2026.05.28.728331; this version posted May 29, 2026. The copyright holder has placed this preprint (which was not certified by peer review) in the Public Domain. It is no longer restricted by copyright. Anyone can legally share, reuse, remix, or adapt this material for any purpose without crediting the original authors.

Supplementary Fig. 4. Avoidance initiation is preserved despite altered motor circuit connectivity.

a, Dimorphic connectivity between interneurons and motor neurons in hermaphrodite and male circuits. Dimorphic connections are highlighted in red.

b, Predicted avoidance index for the dauer circuit with male motor neuron connectivity.
